# Comparison of kinematics and joint moments calculations for lower limbs during gait using markerless and marker-based motion capture

**DOI:** 10.3389/fbioe.2024.1280363

**Published:** 2024-03-12

**Authors:** Tianchen Huang, Mianfang Ruan, Shangjun Huang, Linlin Fan, Xie Wu

**Affiliations:** ^1^ Sports Biomechanics Laboratory, College of Physical Education and Health, Wenzhou University, Wenzhou, China; ^2^ Laboratory of Biomechanics and Rehabilitation Engineering, School of Medicine, Tongji University, Shanghai, China; ^3^ TsingVA (Beijing) Technology Co., Ltd., Beijing, China; ^4^ Key Laboratory of Exercise and Health Sciences, Ministry of Education, Shanghai University of Sport, Shanghai, China

**Keywords:** 3D motion analysis, deep learning, artificail intelligence, joint moment, markerless motion capture

## Abstract

**Objective:** This study aimed at quantifying the difference in kinematic and joint moments calculation for lower limbs during gait utilizing a markerless motion system (TsingVA Technology, Beijing, China) in comparison to values estimated using a marker-based motion capture system (Nokov Motion Capture System, Beijing, China).

**Methods:** Sixteen healthy participants were recruited for the study. The kinematic data of the lower limb during walking were acquired simultaneously based on the markerless motion capture system (120 Hz) and the marker-based motion capture system (120 Hz). The ground reaction force was recorded synchronously using a force platform (1,200 Hz). The kinematic and force data were input into Visual3D for inverse dynamics calculations.

**Results:** The difference in the lower limb joint center position between the two systems was the least at the ankle joint in the posterior/anterior direction, with the mean absolute deviation (MAD) of 0.74 cm. The least difference in measuring lower limb angles between the two systems was found in flexion/extension movement, and the greatest difference was found in internal/external rotation movement. The coefficient of multiple correlations (CMC) of the lower limb three joint moments for both systems exceeded or equaled 0.75, except for the ad/abduction of the knee and ankle. All the Root Mean Squared Deviation (RMSD) of the lower limb joint moment are below 18 N·m.

**Conclusion:** The markerless motion capture system and marker-based motion capture system showed a high similarity in kinematics and inverse dynamic calculation for lower limbs during gait in the sagittal plane. However, it should be noted that there is a notable deviation in ad/abduction moments at the knee and ankle.

## 1 Introduction

In the field of biomechanical research, the motion capture system plays a crucial role in the quantitative analysis of movement. It has been extensively used in sports injury analysis, sports performance improvement, and gait analysis (Quesada et al., 1996; Stone et al., 2013; Fuchs et al., 2019). However, marker-based (MB) systems come with inherent challenges that are hard to circumvent. These include the high cost of cameras, stringent requirements for the experimental environment, and errors in the estimation of joint center positions caused by incorrect placement of reflective markers by operators (Della Croce et al., 1999; Stagni et al., 2000; Mündermann et al., 2006). Additionally, skin movement can introduce noise interference, further complicating the data capture process (Sati et al., 1996; Holden et al., 1997; Fiorentino et al., 2017).

The advancement of artificial intelligence (AI) technology has significantly improved markerless motion capture systems (MMC) (Belić et al., 2019; Lam et al., 2023). MMC have outperformed MB systems in several aspects (e.g., automated capturing, simple operation, and suitability for capturing realistic motion scenarios) (Knippenberg et al., 2017). The Microsoft Kinect, exemplifying monocular MMC, employs RGB and depth images for the acquisition of human movements. Primarily designed for gaming applications, its capability to accurately delineate the subtleties of human motion within a three-dimensional context exhibits significant limitations. Multi-camera MMC may potentially compensate for the deficiencies of monocular cameras in capturing human motion.

Some previous studies have been conducted on the concurrent comparison of kinematic measurements obtained from MMC systems. For instance, Nakano et al. (Nakano et al., 2020) quantified the differences in lower limb joint center positions during walking, jumping, and throwing movements between MB and MMC systems. As indicated by their results, an OpenPose-based multi-camera MMC system could measure the movements of humans with a Mean Absolute Error (MAE) of less than 30 mm for 80% of the joint center positions. Kanko et al. (Kanko et al., 2021a; Kanko et al., 2021b) showed that the MMC system exhibited high accuracy in measuring gait speed, stride length, and stride width with a Root Mean Squared Deviation (RMSD) of less than 25 mm for all joint centers except for the hip joint center, where the RMSD exceeded 30 mm.

Besides kinematic analysis, inverse dynamics analysis serves as a fundamental method for investigating the movement. Inverse dynamics calculation uses kinematic and external force data and human model parameters to determine internal joint forces and moments that are not directly measurable during human movement (Winter, 2009). These parameters provided a quantitative basis for evaluating mechanical loads on the human body during motion (Holder et al., 2020). It is imperative to provide accurate and reliable inverse dynamic parameters to facilitate the widespread use of MMC systems in clinical and research applications. Given the significant role of kinetics data in biomechanical analysis, some researchers have conducted studies on the accuracy of measuring kinetics using MMC systems (Tang et al., 2022; Kanko et al., 2023; Song et al., 2023). For example, Tang et al. (Tang et al., 2022) compared lower extremity joint moments in the sagittal plane during running estimated by MB and MMC systems. As far as we know, however, the differences between these two methods have not been evaluated for lower extremity joint moments in three planes during gait.

This study aimed to compare the difference between the MMC and MB systems for calculating lower limb joint center positions, joint angles, and joint moments during gait.

## 2 Methods

### 2.1 Markerless motion capture system

**FIGURE 1:**
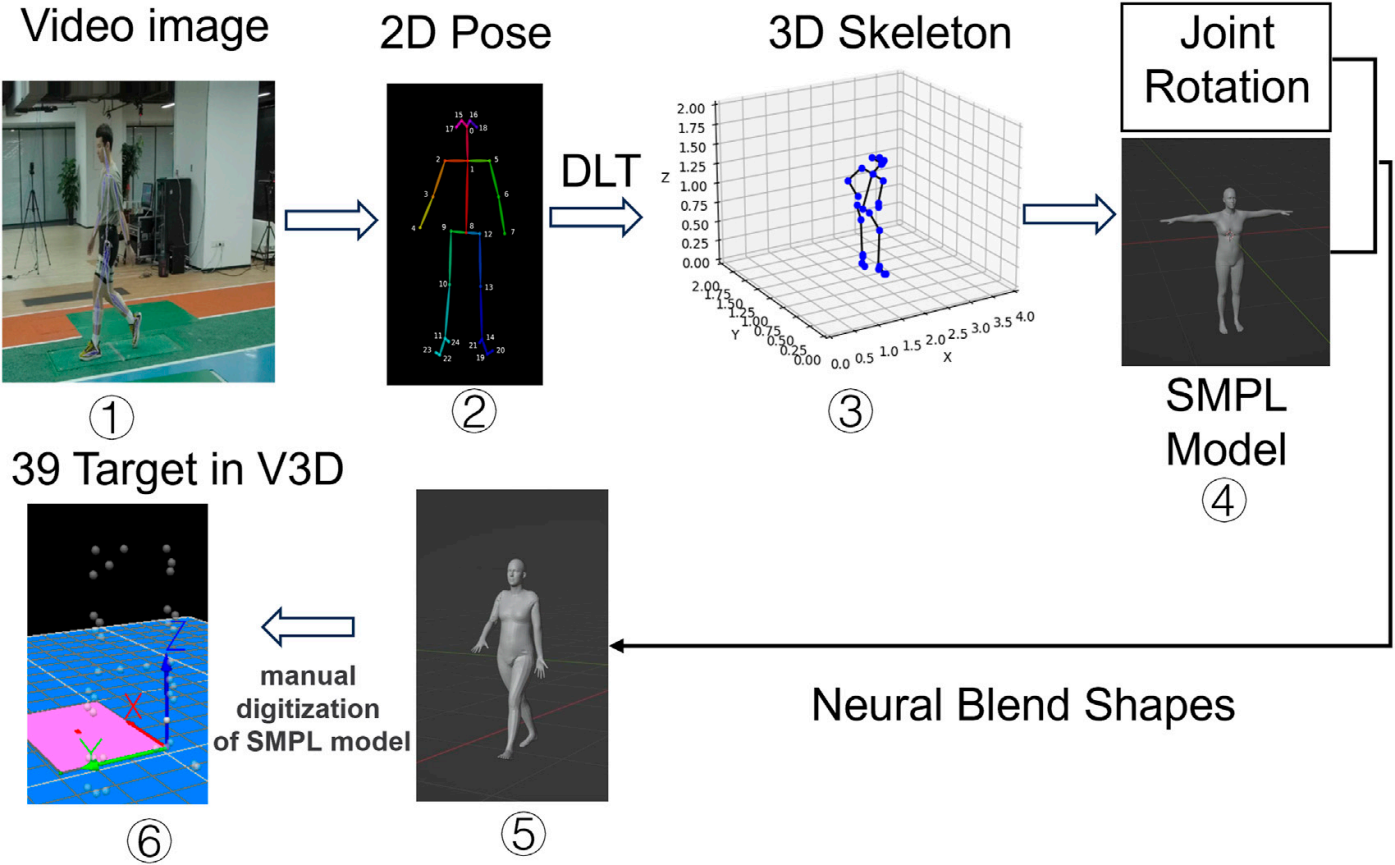
The technology of the MMC system.

Pixmotion (TsingVA Technology, Beijing, China) is a deep learning-based MMC system that captures human motion images and performs 3D human pose estimation (Cao et al., 2017). A deep convolution neural network (Sun et al., 2019) was trained on over 120,000 images of humans in the wild, with 25 human joints manually labeled for each instance. Pix-Motion estimates 25 skeleton joints on 2D images (Cao et al., 2017). The human 3D skeleton is calculated by combining multi-view 2D pose information from 8 cameras, using Direct Linear Transformation (DLT) for camera calibration to map 3D spatial coordinates to 2D image plane coordinates, thus enabling 3D scene reconstruction from 2D images. A Skinned Multi-Person Linear (SMPL) model was fitted, and the human mesh was recovered. Next, a neural blend shapes method (Li et al., 2021) was used to output more than 6,000 vertices on the skin. Finally, 39 human mesh vertices were manually extracted, corresponding to the positions of reflective markers used in the MB system (Figure 1).

### 2.2 Participants

This study was conducted at Peak Sport Science Laboratory in Xiamen, China, with 16 healthy participants (Table 1). The participants provided written informed consent before the commencement of the study, and the institutional ethics committee approved the experimental procedure used in this study. Participants were not engaged in intense exercise within 24 h before the experiment, and no participants had a history of lower limb injury.

**TABLE 1:** Characteristics of participants ( $\bar{x}$ ±SD).

	n	Age/year	Height/cm	Weight/kg
Male	11	30.3 ± 9.3	176.2 ± 6.3	76.6 ± 7.7
Female	5	23.6 ± 1.7	169 ± 1.4	53.2 ± 6.1

### 2.3 Experimental setup and procedure

An MB motion capture system (thirteen Mars 4H (Nokov Motion Capture System, Beijing, China), 4.1-megapixel resolution cameras) and an MMC system (eight Z-CAM-E2 (Intetech, Beijing, China), 120HZ, 8-megapixel resolution) were used to capture the movement synchronously.

**FIGURE 2:**
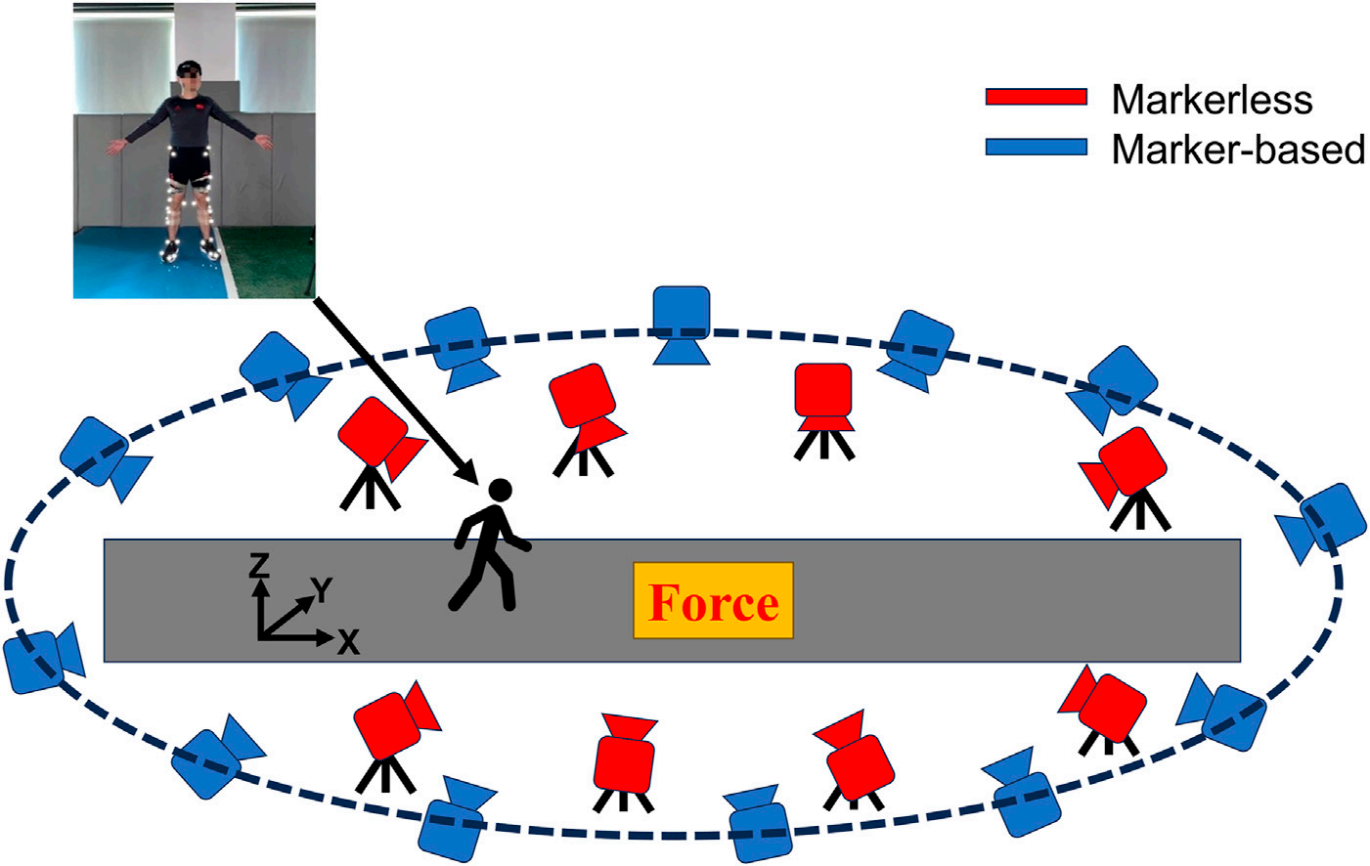
Overhead view experimental setup and markers’ locations on the body.

Both systems were positioned around the runway. The MB cameras were affixed at an elevation of approximately 3 m from the ground, whereas the MMC cameras were placed on tripods at an approximate height of 1.5 m from the ground. The force platform (1200Hz, 9287CA, Kistler Instruments, Winterthur, Switzerland) was placed underground in the middle of the runway (Figure 2). The data collection from both systems and the force platform was synchronized in one click through a self-developed program. A common global coordinate system was built for both systems through the calibration procedure. A static calibration trial for both systems was collected, with the participants standing in the middle of the runway. A total of thirty-nine retroreflective markers were placed as follows: on both the left and right anterior superior iliac spines, iliac crests, greater trochanters of the femur, knee joints, and ankle joints. On each foot, four markers were affixed at the lateral malleolus of the first and fifth metatarsophalangeal joints, the toe, and the heel. In addition, on the lateral sides of both thighs and shanks, four clusters were affixed, with each cluster comprising four markers. Besides, a marker was affixed to the sacrum.

The participants performed three walking trials with at least six steps at a self-selected speed. Participants placed their 4th step on the force platform with the right foot. Retests were required if not done successfully. The initial contact toe-off events were determined using vertical ground reaction force thresholds (>10N for initial contact and <10N for toe-off), while the second contact was determined through kinematic data.

### 2.4 Data analysis

**FIGURE 3:**
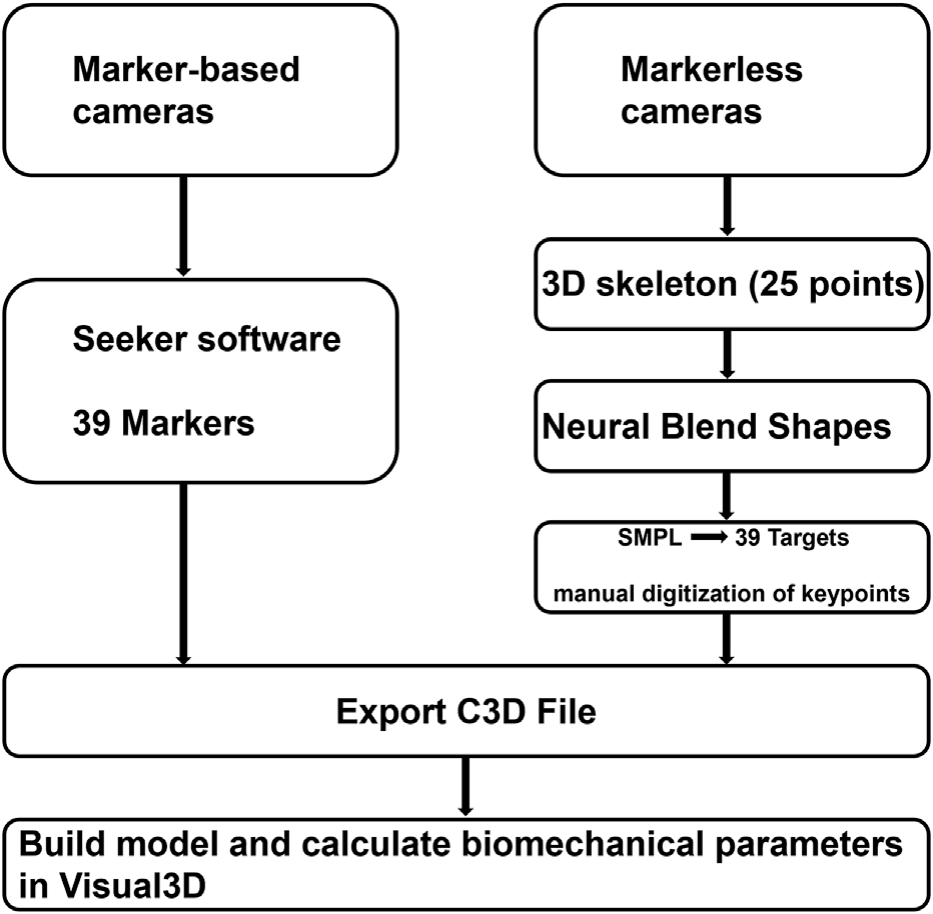
Summary of the workflow.

C3D files generated by MB and MMC systems are input in Visual3D (C-Motion, United States) for inverse dynamics calculations. The lower body kinematic chain in the skeletal model was constrained to have six degrees of freedom (DOF). Figure 3 illustrates the processing workflow following data collection. The raw 3D coordinate data were filtered with a fourth-order Butterworth low-pass filter at 6 Hz, and force data were filtered at 100 Hz. Force-based gait events were used to obtain time-normalized gait cycles. The duration of each stride cycle was scaled to 101 data points.

Joint center positions, joint angles, and joint moments of the lower limb were calculated using the Visual3D models. The right ankle centers were determined by the proximal end of the right shank, the right knee centers by the distal end of the right thigh, and the right hip centers by the proximal end of the right thigh. Joint angles were determined using a Cardan sequence (Grood and Suntay, 1983). The joint moments for both systems were estimated using the inverse dynamics approach (Winter, 2009).

The similarity between the measurement of the two systems was increased with the decrease of the value of RMSD and MAD. The measurement difference of the MMC system was examined using the Coefficient of Multiple Correlation (CMC) (Kadaba et al., 1989) (Eq. 1), MAD, and RMSD (Shcherbakov et al., 2013). The difference of joint moment peak time between MMC and MB was done by performing two-tailed paired t-tests, *p*-value 0.05 was assumed to be significant.

$$\text{CMC}=\sqrt{1-\frac{\sum_{s=1}^{S}\;\sum_{j=1}^{J}\;\sum_{t=1}^{T}\;{\left(X_{\text{sjt}}-\bar{X_{t}}\right)}^{2}/T\left(SJ-1\right)}{\sum_{S=1}^{S}\;\sum_{j=1}^{J}\;\sum_{t=1}^{T}\;{\left(X_{\text{sjt}}-\bar{X}\right)}^{2}/\left(\text{JT}-1\right)}}$$ (1)

S represents the number of motion capture systems (MB and MMC systems); J represents the number of tests; T is the number of time points; $X_{\text{sjt}}$ represents the t time point of the j test of the s system; ${\overset{-}{X}}_{t}$ represents the average of all the curves at time t; $\overset{-}{X}$ is the average of all the curves in the gait. In general, the CMC is classified into four levels:0.75–1 indicates high similarity, 0.5–0.74 indicates moderate similarity, 0.25–0.49 indicates low similarity, and below 0.25 indicates very low similarity (Ferrari et al., 2010).

## 3 Results

### 3.1 Joint center position

**FIGURE 4:**
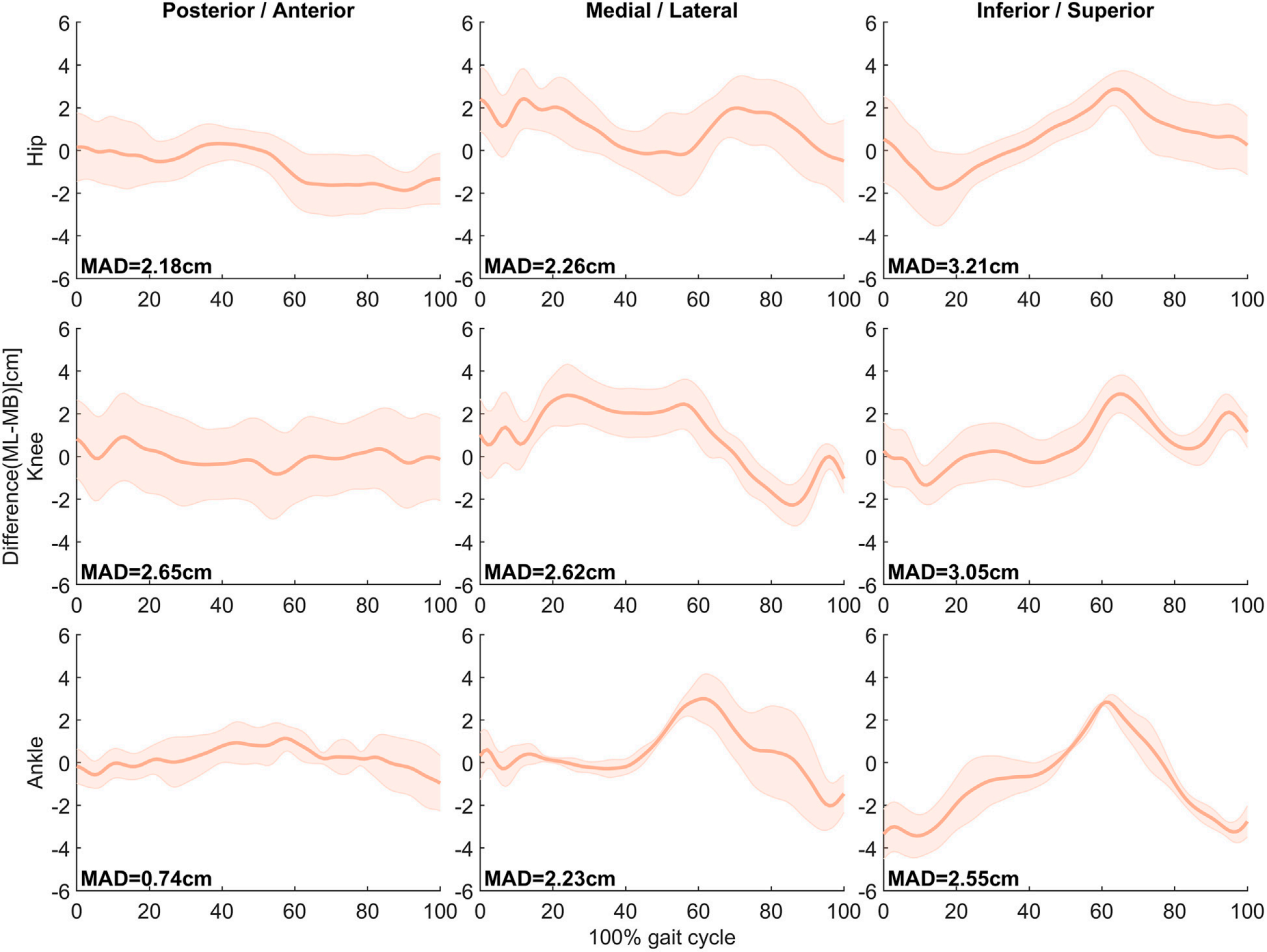
Mean ± SD of differences (MMC–MB) in hip, knee, and ankle joint center positions in the anteroposterior, mediolateral, and internal/external directions during the gait cycle in 16 subjects. MAD is presented in the respective panel.

As shown in Figure 4, the difference in joint centers was the greatest in the superior/inferior direction, 3.21 cm, and the least difference was found at the ankle joint center in the posterior/anterior direction, with MAD of 0.74 cm.

### 3.2 Joint angle

**FIGURE 5:**
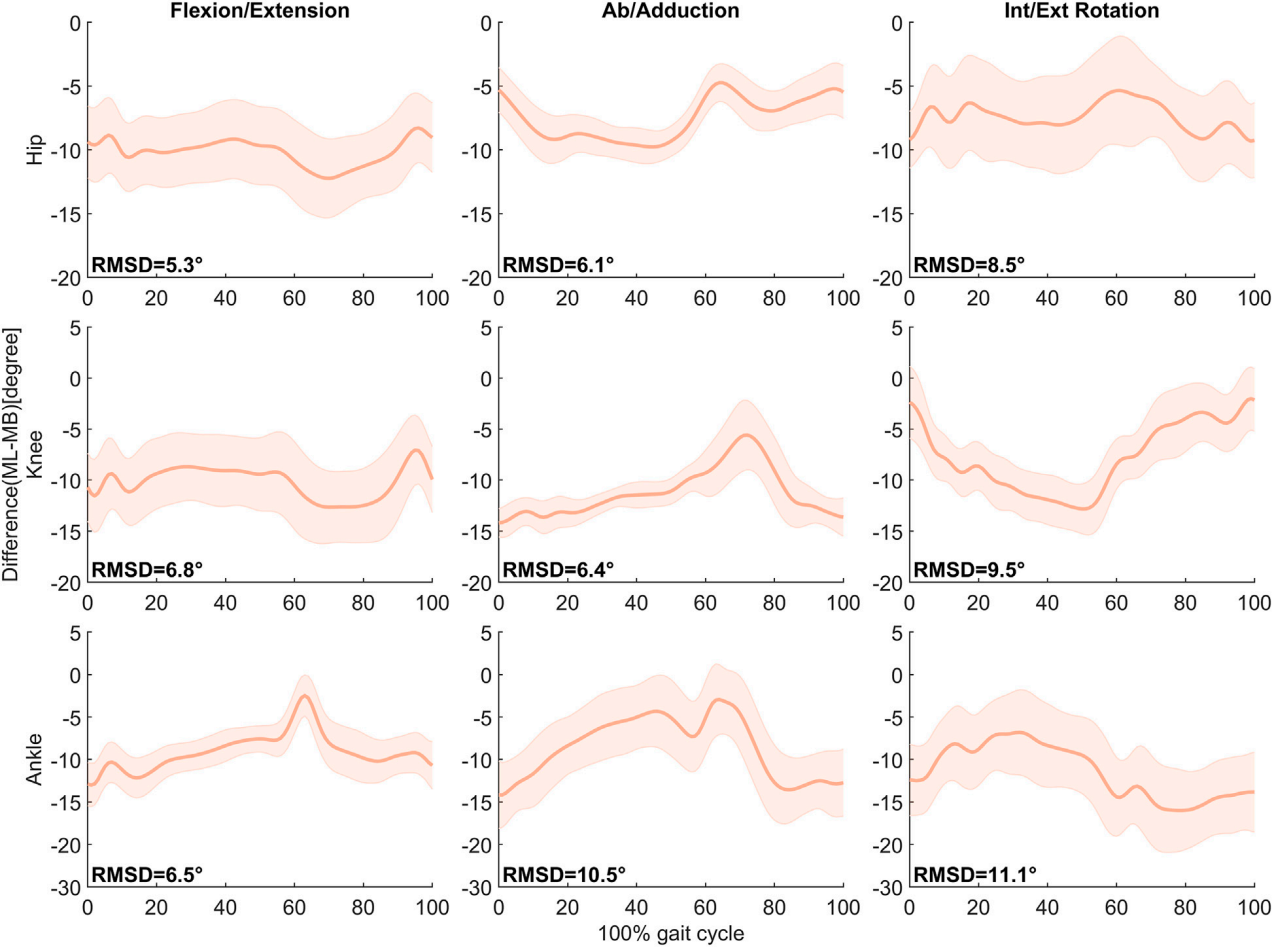
Mean ± SD of differences (MMC–MB) in hip, knee, and ankle joint angles in the Flexion/Extension, Ab/adduction, and Int/Ext Rotation movement during the gait cycle in 16 subjects. RMSD is presented in the respective panel.

As shown in Figure 5, the least difference in measuring lower limb angles between the two systems was found in flexion/extension movement, 5.3°, 6.8°, and 6.5°. The greatest difference was found in int/external rotation movement, 8.5°, 9.5°, and 11.1°.

### 3.3 Joint moment

**FIGURE 6:**
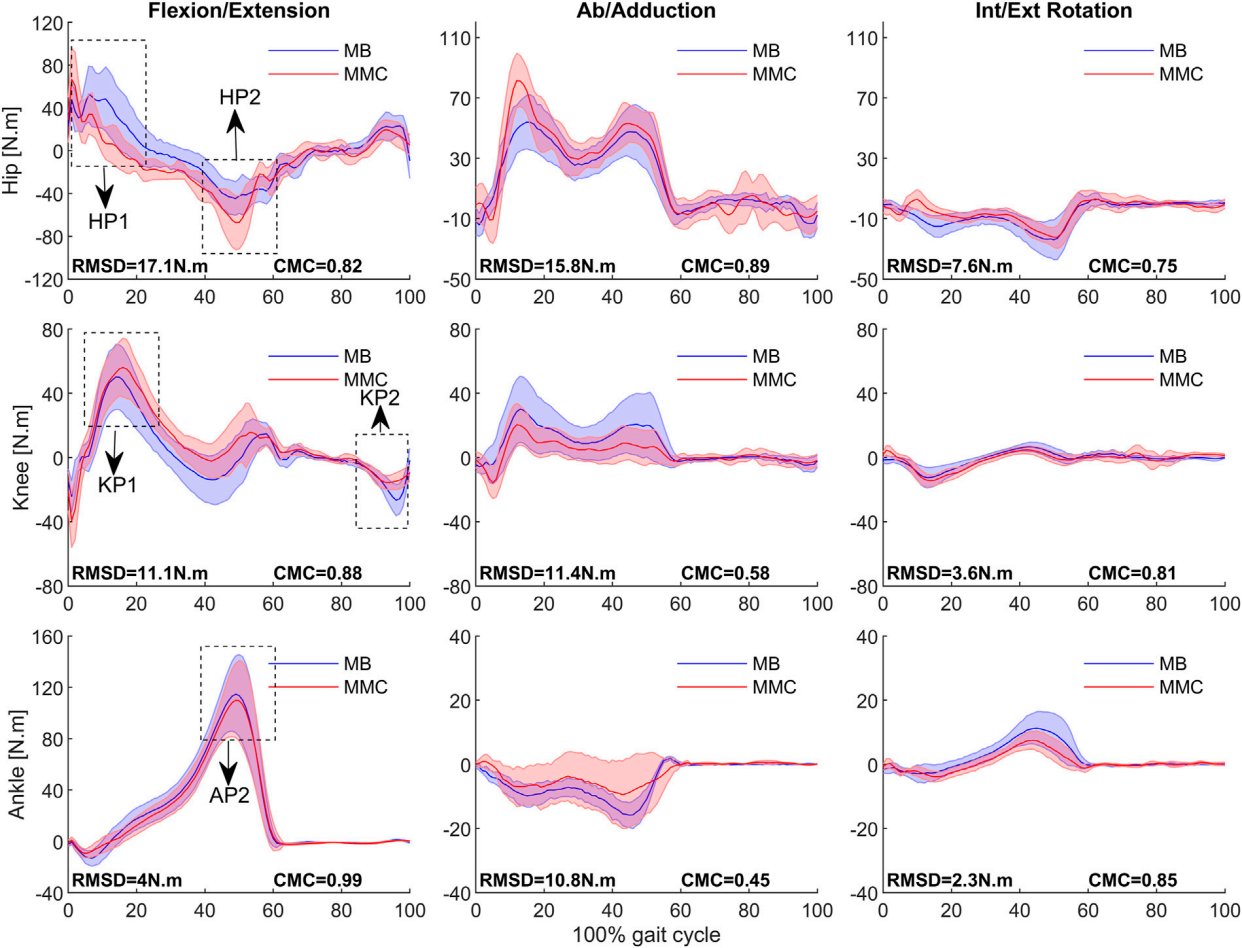
Mean ± SD of MB (blue) and MMC system (red) in hip, knee, and ankle joint moment in Flexion/Extension, Ab/adduction, and Int/Ext Rotation movement during the gait cycle in 16 subjects. RMSD and CMC are presented in the respective panel. HP1 represents the hip’s first peak, HP2 represents the hip’s second peak, KP1 represents the knee’s first peak, KP2 represents the knee’s second peak, and AP1 represents the ankle’s first peak. For the gait phase, 0%–60% is the stance phase, and 60%–100% is the swing phase for the right foot.

Figure 6 shows the mean and variance of three-dimensional joint moments for the lower limb in the two systems. The RMSD between the systems was below 18 N m, with the smallest observed at the ankle joint at 4 N m and the largest at the hip joint at 17.1 N m. The similarity of the curves between both systems was above 0.75, except for the abduction/adduction moments at the knee and ankle joints, where the CMCs were 0.58 and 0.45, respectively. As shown in Table 2, paired t-tests revealed significant differences in the relative timing to peak moments between the MB and MMC systems, specifically for the first and second peak hip moments (HP1, HP2) and the first peak knee moment (KP1). The MMC system reached the peak of HP1 (MB: 5.45 ± 3.76, MMC: 1.35 ± 0.57) more rapidly but demonstrated slower timings for HP2 (MB: 48.5 ± 9.48, MMC: 54.9 ± 6.04) and KP1 (MB: 14.05 ± 1.47, MMC: 15.55 ± 1.88). However, the absolute mean differences observed between the systems for HP1, HP2, and KP1 represented only 4.1%, 6.4%, and 1.5% in the gait cycle. No significant differences were found between the systems when measuring the second peak knee moment (KP2) and the first peak ankle moment (AP1), with the mean difference for AP1 being only 0.2. Refer to Table 2 for more details.

**TABLE 2:** Joint moments peak time as a percentage of gait cycle (mean, SD) for MB and MMC systems in sagittal plane.

	Parameters	MB		MMC		*t*-test
		Mean	SD	Mean	SD	
Moment (%stride Cycle)	Hip first peak	5.45	3.76	1.35	0.57	t = −4.778, *p* < 0.001^a^
	Hip second peak	48.5	9.48	54.9	6.04	t = 2.684, *p* = 0.0147^a^
	Knee first peak	14.05	1.47	15.55	1.88	t = 4.943, *p* < 0.001^a^
	Knee second peak	89.05	9.06	84.25	5.46	t = −1.925, *p* = 0.0694
	Ankle first peak	48.7	1.95	48.9	2.19	t = 0.940, *p* = 0.359

^a^ Indicates significant difference.

## 4 Discussion

This study explored the differences in measuring lower limb joint centers, angles, and moments during gait between MMC and MB systems. It provided a comparative analysis of three-dimensional kinematic and kinetic data derived from both systems.

Compared with the MB system, the MMC system demonstrated a deviation of 0.74 cm in estimating the position of the ankle joint center in the anterior-posterior direction and a 3.21 cm discrepancy in the position of the hip joint center in the vertical direction. The angle differences of the hip, knee, and ankle joints in the sagittal plane were less than 7°. These findings are similar to the differences reported by Nakano et al. (Nakano et al., 2020) and Kanko et al. (Kanko et al., 2021a). Compared to MB systems, the difference in kinematic measurement accuracy of this MMC system is comparable to other MMC systems.

The similarity of waveforms between the joint moment measurements of the two systems was evaluated using CMC, a coefficient that quantifies the simultaneous effects of correlation, gain, and offset. The two systems were highly similar (CMC ≥ 0.75) in calculating lower limb joint moments in the horizontal and sagittal planes. The RMSD < 18 N m was close to the magnitude of difference in lower limb joint moments during weightlifting between both systems (Mehrizi et al., 2017). However, it was much smaller compared to the difference in lower limb joint moments during running between both systems (Tang et al., 2022). Given that we observed CMC greater than 0.8 in the curves within the sagittal plane, further examination of the difference in the relative timing to peak moments can enhance our understanding of the MMC system’s accuracy in gait detection. Regarding the relative timing to peak joint moment, our study found that the two systems showed a high similarity for the knee and ankle joint in the sagittal plane. The greater difference in joint moments and the relative timing to peak moment between the two systems found in a previous study may be due to greater soft tissue artifacts during running (Tang et al., 2022). The difference in joint moments primarily arose from differences in identifying the joint center position because we used the same force data to calculate the joint moment in both systems, and the moments were calculated using the Visual3D software. Important metrics for gait analysis include the relative timing to peak moments and the pattern of the moment curve. Identifying abnormalities in the metrics mentioned above can help trainers and doctors diagnose abnormal gait and perform interventions (e.g., designing targeted training programs and adding customized orthopedic equipment) (Milner, 2009; Harvi et al., 2016). The above-described parameters exhibit little difference in both systems. Although the difference of the MMC system on the abduction and adduction moments needs to be improved, the result on the flexion and extension moments suggests that the MMC system can identify and diagnose abnormal gait on the flexion and extension moments. A considerable number of abnormal gait patterns were reported in flexion-extension moments (Devita et al., 1998; Osada et al., 2021; Stief et al., 2021). For instance, Neckel et al. (Neckel et al., 2008) suggested that gait differences between stroke patients and the healthy population are reported mainly in the support phase, with stroke patients having greater hip extension and knee flexion moments than the healthy population. Consequently, the precise recording of the moment and the timing of moment peaks in the sagittal plane can significantly contribute to the utility of the MMC system in gait analysis applications. Considering the substantial variation in the curves on the frontal plane, we refrained from conducting an analysis of peak moments in that plane. Moreover, while the measurements of rotational moments exhibited commendable CMC values, the loss of information during feature recognition for these moments poses a challenge. Despite our efforts to compensate for this loss by imposing additional constraints in the calculation process, the discrepancies in angles for the rotational moments were still the most pronounced compared to the sagittal and coronal planes, leading to our decision to exclude their computation from the current analysis.

The differences produced by the MMC motion capture system were affected by several factors. Firstly, annotation bias in the training dataset of the MMC system would propagate to the point identification process, increasing the probability of large errors (Martinez et al., 2017). Given that the MMC system employs the SMPL algorithm, the shape parameters necessitate being acquired via image-based methods. However, the current system did not incorporate such data input and thus, relied on the standard body model provided by SMPL. Consequently, the unique body characteristics of the participants were not considered, potentially introducing a certain degree of bias to the generated model of 39 points. Nonetheless, as there were not particularly obese or underweight individuals among the participants, the error is relatively minor. Additionally, due to the utilization of different calibration rods for spatial calibration in the two systems, although manual alignment of the original coordinates was performed, the use of disparate calibration systems still induces a certain degree of error.

The measurement accuracy of the MMC system can be improved in several manners. Firstly, our training dataset was generated through manual annotation, where the margin of error is contingent upon the annotator’s expertise. To enhance the accuracy of the dataset in the later stages, especially for points with larger recognition errors, we plan to provide more specialized training for the standard annotators and employ a multi-person cross-validation annotation approach. Secondly, our current 2D pose tracking system relies on frame-by-frame detection, lacking temporal context between frames. To address this, we plan to develop a neural network that takes video clips as input, aiming to improve joint localization by leveraging temporal correlations. Thirdly, since an SMPL algorithm is employed in this study’s MMC motion capture system, individual differences between different participants may reduce the accuracy of recognition. In subsequent development, personalized data (e.g., the height and size of the subject) should be input into the system by inputting the image information, aiming to increase the SMPL fitting accuracy.

Despite the current limitations of the MMC motion capture system, as AI has been leaping forward, the measurement accuracy of the MMC motion capture system will be further improved, and the application prospects will be significantly expanded. This study indicates the potential for MMC motion capture systems to supplement or replace MB systems, facilitating the extension of human movement data collection from laboratory settings to real-world scenarios, which could significantly impact sports training and sports science research.

## 5 Conclusion

The MMC and MB systems showed a high similarity in kinematics and inverse dynamic calculation for lower limbs during gait in the sagittal plane. However, it should be noted that there is a notable deviation in ad/abduction moments at the knee and ankle.

## Scope statement

This study aimed to provide a reference standard for the application of markerless systems by comparing the difference of marker-based and markerless motion capture systems in calculating the position of lower limb joint centers, joint angles, and joint moments.

## Data Availability

The raw data supporting the conclusion of this article will be made available by the authors, without undue reservation.
